# Early versus delayed umbilical cord clamping on maternal and neonatal outcomes

**DOI:** 10.1007/s00404-019-05215-8

**Published:** 2019-06-15

**Authors:** Yiyu Qian, Xinxin Ying, Peixin Wang, Zhe Lu, Ying Hua

**Affiliations:** Department of Obstetrics and Gynecology, The Second Affiliated Hospital of Wenzhou Medical University, No. 109 Xueyuan Xi Road, Wenzhou, 325027 Zhejiang China

**Keywords:** Delayed cord clamping, Early cord clamping, Maternal outcomes, Prenatal outcomes

## Abstract

**Purpose:**

Policies for timing of cord clamping varied from early cord clamping (ECC) in the first 30 s after birth, to delayed cord clamping (DCC) in more than 30 s after birth or when cord pulsation has ceased. DCC, an inexpensive method allowed physiological placental transfusion. The aim of this article is to review the benefits and the potential harms of early versus delayed cord clamping.

**Methods:**

Narrative overview, synthesizing the findings of the literature retrieved from searches of computerized databases.

**Results:**

Delayed cord clamping in term and preterm infants had shown higher hemoglobin levels and iron storage, the improved infants’ and children’s neurodevelopment, the lesser anemia, the higher blood pressure and the fewer transfusions, as well as the lower rates of intraventricular hemorrhage (IVH), chronic lung disease, necrotizing enterocolitis, and late-onset sepsis. DCC was seldom associated with lower Apgar scores, neonatal hypothermia of admission, respiratory distress, and severe jaundice. In addition, DCC was not associated with increased risk of postpartum hemorrhage and maternal blood transfusion whether in cesarean section or vaginal delivery. DCC appeared to have no effect on cord blood gas analysis. However, DCC for more than 60 s reduced drastically the chances of obtaining clinically useful cord blood units (CBUs).

**Conclusion:**

Delayed cord clamping in term and preterm infants was a simple, safe, and effective delivery procedure, which should be recommended, but the optimal cord clamping time remained controversial.

## Introduction

Compared with ECC, which was usually performed 10–15 s after delivery, DCC of at least 30 s at birth was of great importance for the amount of blood transfused from the placenta to the newborn [1, 2]. Increasing evidence had shown the benefits of DCC in term and preterm infants including the higher hemoglobin levels and iron status, the improved infants’ and children’s neurodevelopment, the lesser anemia, the higher blood pressure, the fewer transfusions, and the lower rates of intraventricular hemorrhage (IVH), chronic lung disease, necrotizing enterocolitis, and late-onset sepsis. Potential disadvantages of DCC are polycythemia, jaundice, and increased requirement of phototherapy, as well as maternal postpartum hemorrhage or the need for maternal blood transfusion.

Recently, the American College of Obstetricians and Gynecologists (ACOG) published a committee opinion that supported DCC in preterm infants. Multiple systematic review and meta-analysis [3, 4] had reported that DCC improved hemodynamic outcomes and reduced hospital mortality, which supported current guidelines recommending DCC in preterm infants. However, for term infants, few reviews demonstrated the advantage of DCC, especially in the acid–base status in umbilical cord blood, hypothermia of admission, and public cord blood bank. Additionally, the timing of DCC was variable, ranging from 30 s to 5 min or to when the cord stops pulsating. Optimal cord clamping time in term and premature neonates remained controversial. Therefore, this article updated and summarized the effect of early versus delayed umbilical cord clamping on maternal and neonatal outcomes in detail.

## The effect of DCC on hemoglobin levels (HB) in term infants

Existing research on DDC had proven its beneficial value not only in term infants but also in premature infants.

For term infants, several randomized controlled trials [5–19] had reported beneficial effects of DCC on infant hemoglobin at birth or at a different duration of follow-ups and demonstrated a subsequent reduction of anemia without unacceptable side effects [5–9]. But one study from Japan reported that the higher hemoglobin values by DCC increased neonatal jaundice in healthy newborns [10].

Ceriani et al. found that DCC of term newborn infants at 1 or 3 min improved venous hematocrit levels measured at 6 h after birth within a physiologic range and decreased the prevalence of neonatal anemia without any harmful effect in newborns or mothers [6]. In a randomized trial from China, 720 term infants were randomized to ECC (< 15 s) and DCC (30, 60, 90, 120, 150, or 180 s, or when the umbilical cord pulsation ceased). The results showed that DCC for at least 60 s could significantly increase neonatal hematocrit levels at 24 h of life, and the mean infant hematocrit increased with the increasing duration of DCC [7]. Likewise, in another prospective randomized controlled trial [8], the term singleton infants were randomized to DCC (≥ 5 min) and ECC(< 20 s), infants with DCC had the higher hematocrit and hemoglobin levels at 24 to 48 h without an increase in adverse effects. The similar results were reported in Libya [9]. In conclusion, these studies [6–9] demonstrated that DCC resulted in the improved hemoglobin levels at birth, which provided evidence of the early hematological advantage of DCC.

A few studies [11–19] on DCC were published in the literature with the different follow-up time ranged from 2 to 12 months, and their results were inconclusive. Delaying the clamping of the umbilical cord until it stopped pulsating improved the hematologic status of the Guatemalan infants at two months after delivery [11]. Interestingly, Ertekin et al. [12] found that DCC up to 90–120 s increased the hemoglobin, hematocrit, and iron levels of infants significantly at the second month, but not at birth. Inversely, in a cohort of newborns with an expected low birth weight in South African, Tiemersma et al. showed that the infant hemoglobin levels at 24 h after birth were significantly higher in the DCC group versus ECC group, while the effects of DCC on the infant hemoglobin levels were not detectable anymore at two months after birth [13].

In addition, a 2-min delay in the clamping of the umbilical cord of normal-weight, full-term infants from Mexico improved the mean corpuscular volume up to 6 months of age [14]. A study done by Nesheli et al. showed that DCC increased the term infants' hemoglobin concentrations and hematocrit at 6 months of age [15]. Furthermore, DCC was effective in improving the hemoglobin levels and in preventing the anemia in 8-month’ [16] or in 8- and 12-months’ infants [17].

However, for the full-term infants born after a low risk pregnancy, there was no significant difference in the infant hemoglobin levels at 4 months of age between DCC (≥ 180 s) group and ECC (≤ 10 s) group [18]. Even though delaying clamping until the cord pulsations ceased could improve the hematological status of term infants living in a highly malarious area before 4 months of age, the beneficial effect of extra red cell mass disappeared by 6 months [19].

Taken together, DCC was a cost free, safe, and effective intervention to reduce anemia and should be implemented in the term infants, especially in resource-poor settings and in developing countries, which might offer a sustainable strategy to reduce early infant anemia risk.

## The effect of DCC on hemoglobin levels in premature infants

Premature neonates are susceptible to anemic problems. Multiple randomized control trials [20–37] had demonstrated that DCC was associated with an increased hematocrit level and an increased neonatal circulating blood, as well as the red blood cell volumes of preterm infants whose gestation ages varied from 24 to 36 weeks among these studies.

In a randomized unmasked controlled trial of infants with a gestational age of 24–28 weeks, Oh et al. [24] found a higher venous hematocrit value at 4 h of age in DCC (30–45 s) versus ECC (< 10 s), which indicated DCC led to an effective placental transfusion at birth. For preterm neonates born between 27 and 31 6/7 weeks of gestation, DCC of 60 s had significant benefits in terms of an increase in hematocrit, compared with ECC (< 10 s) [25]. DCC of 40 s was associated with the higher hemoglobin levels in preterm infants (≤ 29 weeks) at one day after birth [26].

After the implementation of a DCC policy, preterm singleton infants between 24 0/7 weeks and 34 6/7 weeks of gestation, had the increased hematocrits [27, 28]. In preterm infants born between 30 0/7 and 36 6/7 weeks, DCC by 2 min significantly improved the hematocrit values at birth and this beneficial effect continued till at least second month of life [29]. For infants < 32 weeks' gestation or < 33 weeks' gestation, DCC for 30–45 s resulted in an increased hemoglobin concentration at birth [30–32]. Moreover, compared with the 30–45 s DCC group, the 60–75 s DCC group had the higher hematocrits within the first 2 h of life in the whole cohort preterm infants < 32 weeks gestational age, and the higher 12–36 h hematocrit in infants < 28 weeks gestational age [33]. DCC for 60 s in moderate and early late- preterm (MELP) infants born between 32 0/7 and 34 6/7 weeks of gestation was associated with an increased hematocrit at birth [34].

**Fig. 1:**
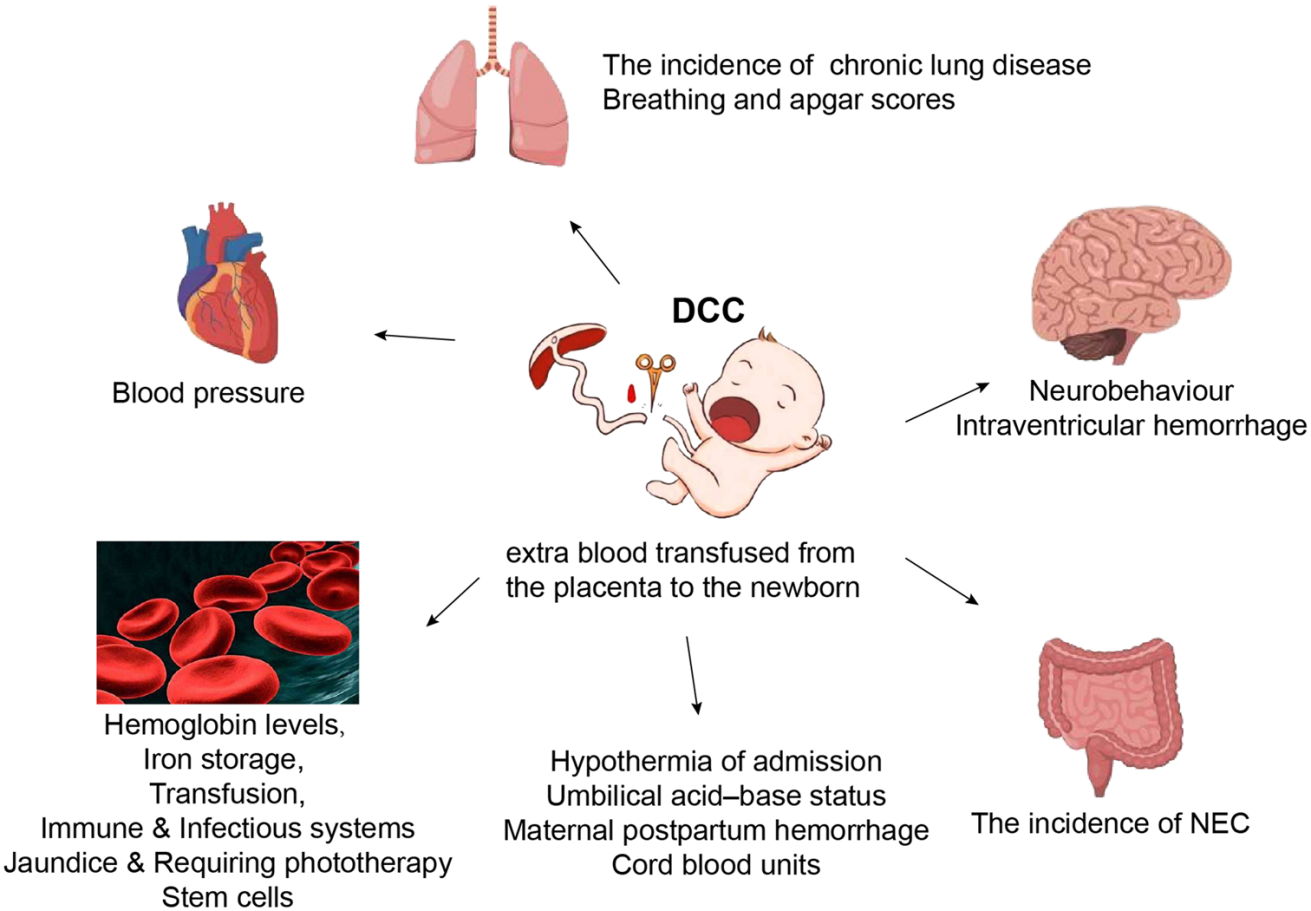
Prenatal phthalate exposure is associated with maternal and fetal outcomes including alteration of gonadal hormones and thyroid hormones levels, disruption of circulating levels of total 25(OH)D, cryptorchidism, hypospadias, and shorter anogenital distance in particularly for male newborns, pregnancy loss, preterm birth, preeclampsia, glucose disorders, growth retardation, allergic diseases, and impaired neurodevelopmental (behavioral, cognitive, and psychomotor and neurobehavioral)

For late preterm neonates between 34 and 36 6/7 weeks gestation, DCC for 120 s had a significantly higher hematocrit level [35], as well as the consistently higher hemoglobin levels than ECC group both at 1 h and 10 weeks of age [36]. In a prospective, masked, randomized, controlled study [37], intention-to-treat analyses revealed that premature neonates born < 35 weeks tended to have a higher hematocrit level in the DCC at 30 to 45 s group versus ECC at 5–10 s group (especially in vaginally delivered neonates). However, only one study [38] reported that no differences were found in the hematocrit level of preterm neonates between DCC for 45–60 s group and ECC within 15 s group, which may be due to the different study design and the small and unevenly matched sample size (Fig. 1).

In summary, most of the studies mentioned above provided evidence that DCC was associated with the benefits of the higher hemoglobin levels, blood volume, and red blood cells in the preterm infants. Clinicians should advocate for the implementation of DCC as a part of the resuscitative process for preterm neonates. Future studies with long term follow-up for the infant hemoglobin levels are required for firm recommendations.

## The effect of DCC on iron storage in infants

Iron deficiency anemia is a major public health problem in young children worldwide, and is associated with poor neurodevelopment even in preschool and school children. Several studies [14, 17–19, 29, 36, 39–42] had reported the effect of DCC on iron stores at the different follow-up time in term and preterm neonates.

For normal weight and full-term infants in Mexico, Chaparo et al. showed DCC of 2 min improved the iron status at 6 months of age compared with ECC of 10 s [14]. The similar result was shown in term infants from a malaria-endemic region [19]. The ferritin concentration was significantly higher, and the iron deficiency anemia at 4, 8, and 12 months of age was significantly less prevalent in the DCC group [17, 18].

For term infants born to anemic mothers (Hb < 10 g/dl), a randomized controlled trial demonstrated that the mean infant ferritin and hemoglobin levels at 3 months were significantly higher in the DCC group than in the ECC group [42]. Especially for vulnerable small for gestational age (SGA) infants born at ≥ 35 weeks, who are those with birth weight less than 10th centile for the gestation and sex, Chopra et al. systematically evaluated the effects of DCC on iron stores and found that the median serum ferritin levels at 3 months were higher and fewer infants had iron deficiency in DCC group compared to ECC group [40]. Interestingly, in a prospective observational study, Andersson et al. observed that iron stores at 4 months in infants born after elective cesarean section with DCC for 30 s were comparable to those in vaginally born infants subjected to DCC for ≥ 180 s, and were improved compared with vaginally born infants subjected to ECC for ≤ 10 s [41].

Other studies [29, 39] were conducted in preterm neonates to add to the existing evidence on the benefits and the safety profile of DCC. In preterm neonates of 30–33 weeks of gestation, DCC had resulted in improved ferritin levels at discharge in comparison to ECC, but this benefit did not sustain till 3 months of postmenstrual age [39]. In preterm infants born between 30 and 36 6/7 weeks of gestation, DCC of beyond 2 min was associated with the higher ferritin at 6 weeks of age [29]. However, Ultee et al. [36] observed that DCC did not affect the ferritin level at 10 weeks of age in infants born between 34 and 36 weeks’ gestation.

In conclusion, DCC was a well-established strategy to improve iron stores in term and preterm infants and seemed to benefit infants even in regions with a relatively low prevalence of iron deficiency anemia. Future researches in this field should be directed at assessing the long-term effects of DCC on iron status in communities with higher rates of iron deficiency anemia.

## The effect of DCC on neurobehavior in infants

Iron deficiency in infant will impair neurodevelopment [43]. The effects of DCC on the neurobehavior of preterm infants remained unclear. One study from Nepal demonstrated that DCC in late preterm and term infants had reduced infant anemia, and thereby improved infants’ and children’s neurodevelopment [44]. A brief delay of 30–60 s in cord clamping was beneficial in improving short-term neurobehavioral outcome of late preterm infants (34–36 weeks), who showed a higher score in both motor development vigour (MDV) and alertness orientation at 37 weeks post-conceptional age [45].

Andersson et al. conducted a series of follow-up study about the effect of DCC on neurodevelopment in infants from 4 months to 4 years [46–48]. Compared with ECC (< 10 s), DCC for 180 s or more did not affect overall neurodevelopment in full-term infants up to 4 months of age, but had higher scores in the problem-solving domain and lower scores in the personal–social domain [46]. Although DCC improved hematological status in the newborn period and the iron status at age 4 months, it did not affect the iron status or the neurodevelopment at age 12 months in the same study population of healthy term-born infants [47]. In addition, similar overall neurodevelopment and behavior among 4-year-old children were shown between DCC and ECC [48].

Interaction analysis showed that DCC lowered Ages and Stages Questionnaire (ASQ) scores in girls and increased them in boys, which indicated that sex may influence the effects of DCC on the infant development [47]. In boys, DCC was associated with the higher scores on several tests including the processing speed quotient, the bicycle trail task, and the ASQ fine motor and personal–social domains, but no differences were shown in girls [48]. In consistent, Mercer et al. observed that a brief delay (30–45 s) in cord clamping was associated with higher Bayley Psychomotor Developmental Index scores for very low birth weight (VLBW birth weight < 1500 g) male infants at 7 months corrected age, which suggested that DCC could be protective of VLBW male infants against motor disability [49].

## The effect of DCC on blood pressure in infants

The higher mean blood pressure (BP) may contribute to an improved hemodynamics and organ perfusion in newborns, which may benefit from an effective placental transfusion and extra blood volume from DCC.

Katheria et al. detected that 5-min DCC was associated with greater BP at 12 h of life in the term infants [50]. In preterm neonates between 27 and 31 6/7 weeks of gestation, DCC for 60 s had higher blood pressure [25]. DCC for 45 s in preterm infants (< 33 weeks) was associated with less blood pressure support [32]. In addition, in a randomized clinical trial of DCC (30–45 s) versus ECC (5–10 s), intention-to-treat analyses revealed that preterm infants born between 24 and 32 weeks of gestation in the DCC group had higher initial mean blood pressures [51]. Another intention-to-treat analyses from Kugelman et al. detected that the group of DCC for 30–45 s in premature neonates (< 35 weeks) tended to have higher initial diastolic BP [37].

However, several studies [24, 30] showed null effect of DCC on the BP in the preterm infants. In a prospective case–control study, when controlling for key clinical variables, DCC between 30 and 45 s in infants born before 32 weeks of gestation was not associated with a difference in mean arterial blood pressure [30]. There was no difference in hourly mean arterial blood pressure during the first 12 h of life in infants with a gestational age of 24–28 weeks between ECC (< 10 s) group and DCC (30–45 s) group [24]. Furthermore, in VLBW preterm infants who were randomized to either 20 s or 45 s of cord clamping, no difference was observed in blood pressure [52].

To our knowledge, no studies had reported that DCC was associated with lower initial mean blood pressures in preterm infants. It is difficult to make a decision of DCC for newborns requiring for resuscitation. Future studies should be undertaken for assessing the effect of DCC on BP in preterm neonates who require immediate resuscitation.

## The effect of DCC on the need of transfusion in infants

As a method of autologous transfusion of blood, DCC resulted in an increase in blood volume and red blood cells in neonates. Thereby, whether the intervention with DCC in preterm infants would reduce the need for blood transfusion was concerned recently.

Some studies found that DCC in infants born before 33 weeks of gestation was not associated with the total number of transfusions [30, 32]. Similarly, in a randomized, controlled unmasked trial in which women in labor with singleton fetuses < 32 weeks' gestation, there were no significant differences in the amount of blood loss and transfusion between ECC (5–10 s) and DCC (30–45 s) groups [31]. DCC for 30 s did not decrease the need for blood transfusion among preterm neonates born between 24 and 34 weeks of gestation [28].

VLBW infant is a special group who is extraordinarily fragile and often requires blood transfusions. The implementation of DCC led to a reduction in the number of blood transfusions given to the VLBW infants [24, 53, 54]. It appeared that in very preterm infants, extra placental transfusion achieved by DCC was a relatively effective and safe intervention that could lead to a reduction of red cell transfusions.

## The effect of DCC on the incidence of intraventricular hemorrhage in infants

Intraventricular hemorrhage (IVH) occurs when a cerebral hemorrhage expands into the brain ventricular system via a variety of mechanisms [55]. Few groups studied the effect of DCC on the IVH in full-term infants due to the low incidence of IVH. Preterm neonates are a high-risk demographics of IVH. Some studies [33, 56] showed that DCC in preterm infants did not alter the incidence of IVH, while others [27, 28, 31, 38, 57] reported that this intervention reduced the risk of IVH.

One randomized controlled trial found that there were no differences in rates of IVH in fetuses (≤ 32 weeks gestation) between DCC (30–45 s) and ECC (< 10 s) groups [56]. The same results were showed in an observational study that compared the 30–45 s DCC with the 60–75 s DCC [33].

However, a significant reduction was noted in the incidence of IVH in the DCC (45 s) cohort compared with the historic control group [57]. After the implementation of a DCC policy, preterm singleton infants born between 24 and 34 6/7 weeks of gestation had a decrease in the incidence of IVH [27, 28]. In addition, VLBW infants in the DCC group (30–45 s) had less IVH during the first 28 days, especially for male infants [31]. These clinical trials suggested that DCC in preterm and VLBW infants was efficacious in reducing the risk of IVH [27, 28, 31, 57].

In summary, although these results provided support for a DCC policy for preterm infants for the prevention of neonatal IVH, the effect of extending the timing of DCC on IVH is needed to explore in further studies.

## The effect of DCC on the incidence of necrotizing enterocolitis and chronic lung disease in infants

Several studies [26, 28, 31–33, 58] compared the effects of early versus delayed cord clamping on necrotizing enterocolitis and chronic lung disease in preterm infants.

The incidence of necrotizing enterocolitis in preterm neonates (24–34 weeks of gestation) had no statistically significant difference between the ECC (5 s) group and the DCC (30 s) group [28], which was consistent with the trial with singleton fetuses < 32 weeks' gestation between the ECC (5–10 s) and the DCC (30–45 s) groups [31]. But Aziz et al. observed that DCC for 45 s was instituted for babies born between 28 and 32 weeks’ gestational age, who had lower rates of necrotizing enterocolitis [32].

In a large, multicenter, randomized trial, DCC (≥ 60 s) did not affect the incidence of chronic lung disease at 36 completed weeks of postmenstrual age in infants born alive before 30 weeks of gestation [58]. Furthermore, increasing DCC duration from 30–45 to 60–75 s had no effect on chronic lung disease in preterm infants < 32 weeks gestational age [33]. However, an observational study [26] reported that DCC for 40 s was associated with an increase in chronic lung disease for infants ≤ 29 weeks, though with no significance. But all of the studies were still hard to illuminate the effect of DCC on necrotizing enterocolitis or chronic lung disease because of the low incidence and few evidences.

## The effect of DCC on immune and infectious systems in infants

The umbilical cord blood also contains various stem cells that play an essential role in repairing tissue and building immunocompetence [59]. DCC with effective placental transfusion improved hemodynamic stability, thus reducing the vulnerability of infants to inflammatory processes [60]. Based on this analysis, several organizations had studied the effects of DCC on the immune and infectious systems during the neonatal period.

A prospective, masked, randomized, controlled, single-center study reported that DCC (30–45 s) did not affect the immunologic or the infectious status of infants born at < 35 weeks during the neonatal period compared with ECC (5–10 s). All infectious parameters including the complement levels (C3 and C4) and the levels of the immunoglobulins (IgG and IgM) were comparable between the ECC and DCC groups [61].

Late-onset sepsis (LOS), defined as blood culture positive in infants > 72 h of age, may be a result of immunocompromise due to loss of protective stem cells. Some trials [21, 31] had shown that DCC in preterm infants resulted in lower risks of LOS. But other trials [27, 56] found that DCC did not alter the incidence of LOS in preterm infants. Furthermore, significant differences were not found in the rates of LOS between the 60–75 s DCC and the 30–45 s DCC groups [33].

An alloimmune disorder due to maternal and fetal blood type incompatibility, is associated with fetal and neonatal complications related to red blood cell (RBC) hemolysis. A recent single-center, retrospective cohort study by Garabedian et al. highlighted that DCC may be a safe, effective, cost-free strategy to prevent the need for postnatal exchange transfusions in cases of RBC alloimmunization [62]. The authors demonstrated that DCC improved the hemoglobin level at birth and longer delay between birth and first transfusion with no severe hyperbilirubinemia. Infants with red blood cell alloimmunization benefit from DCC due to a decreased postnatal exchange and top-up transfusions needs. Thus, DCC with duration of 30 s in infants at risk for red blood cell alloimmunization neonatal anemia is recommended. It will be interesting to evaluate the long-term effects of DCC in this population.

## The effect of DCC on hypothermia of admission in infants

With the implementation of a DCC policy, several researches found that this procedure could affect the incidence of hypothermia in preterm infants. Concerns have been raised regarding the risk of hypothermia during the procedure of DCC.

In the randomized controlled trial [25] of preterm neonates between 27 and 31 6/7 weeks of gestation, DCC (60 s) was associated with less hypothermia of admission in NICU compared to ECC (10 s). Infants ( ≤ 29 weeks of gestation) who received DCC (45–60 s) had the higher admission axillary temperature [38], and babies of < 33 gestational weeks had the fewer hypothermia ( < 36.3 °C) after DCC for 45 s [32]. Furthermore, extending DCC duration from 30–45 to 60–75 s decreased hypothermia on admission in preterm infants < 32 weeks gestational age, but not in preterm infants < 28 weeks gestational age [33].

Some researches [52, 57, 63] found no difference in incidence of hypothermia in preterm infants of different gestational age who received DCC from 45 to 75 s. One study [52] reported that there were no significant differences in temperature on admission in 40 infants (< 33 gestational weeks) who were randomized to either 20 s or 45 s of late cord clamping. Another small pilot study in which 29 infants < 33 weeks’ gestation received assisted ventilation during DCC observed a similar rate of NICU admission hypothermia compared with ECC [63]. Also, no significant differences in admission temperature between the DCC and ECC groups were found in infants born at ≤ 32 weeks’ gestational age [57], and in VLBW infants [54].

However, the DCC pilot group during elective cesarean deliveries might experience more cold stress or hypothermia (admission temperature ≤ 36.2 °C) compared to the historical controls group [64]. But there was no difference in prevalence of more severe hypothermia of admission temperature < 36.0 °C [64].

In conclusion, regardless of it seems to DCC could prevent the hypothermia, more aggressive prevention of infant heat loss may be warranted. Further clinical studies are needed to determine the effects of DCC duration on hypothermia of very preterm infants.

## The effect of DCC on breathing and Apgar scores in infants

For preterm infants, due to immature cardiopulmonary systems, the respiratory transition from a fetus to a neonate at birth usually requires some form of assistance. Currently, to avoid a delay in initiating resuscitation, the practice of DCC in preterm infants has not been adopted widely, particularly for infants who are not vigorous at birth. Establishing ventilation in the infant before the umbilical cord clamped and the impact of DCC on breathing in preterm infants remained under study.

Aziz et al. [32] had reported that DCC was associated with a trend toward lower 1-min Apgar scores, but not 5-min Apgar score. But most of the studies [24–26, 32, 57, 62, 63] provided the evidence that there were no significant differences in Apgar scores and the frequency of events during delivery room resuscitation or the number of infants requiring resuscitation in preterm infants between ECC group and DCC group. Despite delaying resuscitation briefly (around 40 s), Apgar scores and other resuscitation variables in preterm infants were not different between ECC group and DCC group [26], as well as no significant difference in breathing at birth and the need for positive pressure ventilation or intubation in the delivery room [26, 32, 52].

Interestingly, Fenton et al. [38] found that infants of more than 29 weeks’ gestational age who received DCC had significantly higher 1-min Apgar scores but not 5-min Apgar scores, which was in contrast with the results from Aziz et al. [32] but this effect was not found in infants of 29 or less weeks’ gestational age. In a small pilot study, Winter et al. reported that resuscitation of assisting ventilation in infants < 33 weeks’ gestation during 90 s of DCC was feasible and safe, and the median Apgar scores in units that receivedassisted ventilation were comparable to the control group [63]. Infants who are thought to need resuscitation, receiving DCC may benefit the most, perhaps by increasing the accepting reservoir for placental blood through decreasing pulmonary vascular resistance. Thus, many researchers advocated clinician for the implementation of DCC as part of the resuscitative process for preterm neonates.

Delayed cord clamping yielded a greater blood volume and increased pulmonary circulation and cardiac output, allowing greater oxygen delivery to the tissues and decreased need for resuscitation interventions. Published studies [34, 37, 54, 57] reported consistent results that DCC in very preterm infants was well tolerated and the majority established spontaneous respiration while DCC was occurring and breathing benefits from DCC. Additionally, Katheria et al. evaluated 1 min versus 5 min of DCC in term newborns at risk for resuscitation and demonstrated that 5-min DCC could be accomplished safely without compromising the ability to perform resuscitation [50].

Katheria et al. compared DCC-only group (without assisted ventilation, only with dried and stimulated by gently rubbing the back if apneic) with V-DCC group (initial continuous positive airway pressure, with addition of positive pressure ventilation if needed) and found that the onset of breathing was similar between DCC-only group and V-DCC group, and V-DCC of at least 60 s was feasible but did not lead to any measurable clinical improvements immediately after delivery [65]. One unexpected outcome was the high number of infants that began to establish respirations during DCC. The provision of gentle tactile stimulation during DCC may hasten the establishment of spontaneous respirations and provide a similar placental transfusion compared to early continuous positive airway pressure with or without the addition of positive pressure ventilation [65]. It was a striking observation that 95% of babies (< 33 weeks’ gestation) enrolled to DCC initiated spontaneous respirations within 45 s [32], but it is worth noting that infants who did not breathe during DCC had worse outcomes [26].

Chiruvolu et al. [57] reported that the incidence of intubation in delivery room, respiratory distress syndrome, and surfactant administration were significantly lower in the DCC cohort compared with the historic cohort. Then in 2018, they demonstrated similar results that fewer infants were admitted to neonatal intensive care unit (NICU) on respiratory support, the incidence of respiratory distress syndrome was significantly lower and respiratory transition at birth was better in the DCC cohort compared with the historic cohort in infants born between 32 0/7 and 34 6/7 weeks gestation [34]. Increasing DCC duration from 30–45 to 60–75 s was associated with a 50% reduction in need for delivery room intubation and a significant reduction in surfactant therapy, intubation in the first 24 h of life, and any intubation during the NICU stay [33]. Regarding VLBW infants, DCC of 30–45 s or 60 s was associated with less need for respiratory support and surfactant therapy [37, 54].

Taken together, DCC in very preterm infants who need initiating resuscitation appeared to be safe, feasible, and effective. Even though the complexity of providing supportive interventions with the umbilical cord intact, these published studies [24–26, 32, 57, 62] recommend DCC for newborns who require resuscitation at birth. However, implementing DCC for preterm deliveries was complex, involved obstetric and neonatal team training, and required modification of timing of resuscitation interventions to the timing of cord clamping. An important consideration is whether resuscitation interventions would be beneficial during a delayed cord clamping. Therefore, further clinical studies are needed to optimize the timing and technique of DCC and to assess the impact of this potentially valuable procedure on longer term evaluation of morbidity and mortality of the preterm infants, especially in those infants who fail to breathe during transition and DCC.

## The effect of DCC on jaundice and requiring phototherapy in infants

At birth, DCC allowed placental blood transfusion. Hyperbilirubinemia, hypervolemia, polycythemia, and hyperviscosity syndrome were frequently mentioned adverse effects of placental transfusion, which had hindered the adoption of DCC. Evidence from some studies [6, 8, 9, 15] had shown that DCC did not lead to a significant difference in neonatal polycythemia, hyperbilirubinemia or the number of infants requiring phototherapy. Other studies [5, 10, 37, 38, 66], however, showed DCC increased rates of hyperbilirubinemia, polycythemia, and transient tachypnea in the newborn.

In term infants, no significant difference in polycythemia, hyperbilirubinemia and tachypnea was found between the DCC (50–60 s) and ECC groups [15]. Compared with ECC, DCC of 5 min or after cord stopped pulsating did not affect total serum bilirubin levels at 24 h, clinical jaundice or plethora and symptomatic polycythemia in term infants born to American mothers [8] and Libyan mothers [9]. Plasma bilirubin values as well as hyperbilirubinemia rates in term newborns were almost identical in the three groups (within the first 15 s, at 1 min, or at 3 min after birth) [6], which goes along with other authors’ observations [8, 9, 15].

Furthermore, no significant differences were seen in highest bilirubin level in babies born at less than 33 weeks' gestational age [32], and in pathological jaundice or polycythemia in neonates born 34–36 weeks' gestational age [36]. For VLBW neonates, DCC for 45 s or 60 s was also not associated with a change in peak bilirubin [20] and an increase in the utilization of phototherapy [54]. Even if more neonates needed phototherapy after DCC, initial bilirubin levels and extent of phototherapy did not differ in preterm infants [23].

In addition, despite successful placental transfusion, hyperbilirubinemia and hyperviscosity were not observed in South African neonates with expected low birth weight [13]. Garabedian et al. [62] found that DCC resulted in no increase in severe hyperbilirubinemia in red blood cell alloimmunization. DCC in cesarean section has no significant effect on the highest bilirubin after birth, the occurrence of neonatal hyperbilirubinemia or requirement of phototherapy for neonatal jaundice [67, 68]. Taken together, not only in term newborns but also in preterm newborns, DCC was not associated with severe hyperbilirubinemia and increases in jaundice requiring phototherapy regardless of delivery by vaginal or cesarean.

On the contrary, for preterm infants, several studies [37, 38, 66] observed a trend toward higher peak serum bilirubin levels in the DCC group than in the ECC group. Neonates in the DCC group required a longer duration of phototherapy and had a trend towards higher risk of polycythemia [29]. For term newborns, serum bilirubin after 6 h of birth was slightly higher in the DCC group versus ECC group [5]. The rate of jaundice requiring phototherapy was also elevated by DCC in healthy Japanese newborns [10]. Accordingly, DCC improved the hematological status of the babies in early infancy with a risk of polycythemia and jaundice requiring phototherapy.

To summarize, due to these inconsistent results among the studies [5, 10, 13, 37, 38, 67], a more comprehensive appraisal of delayed cord clamping is warranted.

## The effect of DCC on maternal postpartum hemorrhage

To reduce postpartum hemorrhage and the need for blood transfusions to the mother, ECC was recommended [69]. However, some studies [6, 14, 19, 68, 70, 71] had reported no association between DCC in term and preterm infants, and maternal risk of postpartum hemorrhage, blood loss at delivery, or the need for blood transfusion. These trials were diverse in measuring blood loss (visual estimation versus measuring jar), in the mode of delivery (vaginal versus caesarean section), in the duration of DCC from ≥ 30 s to 3 min, and in single or multifetal gestation.

In term infants, several randomized controlled trials [6, 14, 19, 68, 70, 71] evaluated the effect of DCC on maternal blood loss. In the study of 358 mother–infant pairs from Mexico, DCC for 2 min in normal weight and full-term infants was not associated with the increased estimated maternal bleeding at delivery assessed by the physicians [14]. No differences were found in the midwives’ assessment of maternal bleeding and maternal hemoglobin on average 16-h postpartum between DCC and ECC [19]. Limitation of the two studies [14, 19] was that they were not able to quantitatively measure maternal blood loss.

Other studies [6, 70, 71] had observed similar results from more objective methods of measuring maternal blood loss during the third stage of labor. The trial of term neonates born from mothers without complications in Argentina found no significant differences in maternal postpartum hemorrhage and maternal hematocrit level 24 h after birth among three groups of cord clamping within the first 15 s, at 1 min, or at 3 min after birth [6]. Similarly, 97 healthy pregnancies at term and vaginal delivery were randomized assigned to ECC group (< 10 s) or to DCC group (2 min). A blood test at 48 h post-delivery to evaluate maternal postpartum blood loss showed no statistical differences among hematological parameters [70]. When the term deliveries were randomized to DCC (≥ 180 s, *n* = 193) or ECC (≤ 10 s, *n* = 189) in Swedish county hospital, the differences in the maternal postpartum hemorrhage between the DCC and ECC groups were small and non-significant [71]. Taken together, these results [6, 14, 19, 70, 71] suggested that DCC did not have a significant effect on maternal postpartum hemorrhage.

Recently, research [64, 68] on the optimal time to clamp the cord during cesarean deliveries had been conducted. In the randomized controlled trial of 158 women undergoing antepartum lower segment caesarean section (LSCS) between 37–39 weeks of gestation, who was divided into three groups [The umbilical cord of the baby was clamped at < 15 s (*n* = 52) or between 60–75 s (*n* = 52) or between 120–135 s (*n* = 52)], there was no significant differences in post-operative hemorrhage among the three groups [68]. Data from a pilot safety study suggested that DCC for 2 min did not increase the risk of excessive maternal blood loss in elective, term cesarean [64]. Therefore, they [64, 68] believed that DCC during antepartum LSCS was feasible and safe, and there should be no hesitation in implementing this procedure routinely.

Particularly, in a retrospective cohort study of pregnant women with multiples, Ruangkit et al. [72] did not find an increased rate of maternal blood loss, bleeding complications, maternal blood transfusions, and post-delivery decrease in hematocrits, when DCC (> 30 s) was performed in multiple pregnancies compared to ECC (< 30 s). However, another retrospective cohort study [73] of deliveries occurring at < 34 weeks showed that DCC of 30 s of multiple gestation in cesarean delivery increased maternal estimated blood loss in regression analyses without the result of an identifiable increase in adverse maternal outcomes.

In conclusion, DCC did not appear to increase the risk of excessive maternal postpartum hemorrhage and was a feasible method from an obstetric perspective. More evidence from randomized trials was needed to assess safety and feasibility of longer periods of DCC regarding what can be considered to be the best time to clamp the cord, particularly in cesarean delivery, preterm, and multiple pregnancies.

## The effect of DCC on umbilical acid–base status

The acid–base status in umbilical cord blood at birth reflects the newborn’s aerobic and anaerobic intrauterine metabolisms and is an objective retrospective measure of fetal exposure to hypoxia during labor. Arterial umbilical cord blood gas analysis (BGA) is an essential criterion to define neonatal encephalopathy and cerebral palsy due to an intrapartum cause. It is well known that one main advantage of ECC is facilitation of blood gas sampling from the umbilical cord. Thus, information on possible BGA changes after DCC is scarce.

In a prospective observational study of vaginally delivered term newborns, Wiberg et al. [74] sampled umbilical cord arterial and venous blood at delivery (*T*_0_), at 45 s (*T*_45_), and 90 s (*T*_90_). They reported that persistent cord pulsations with DCC at birth resulted in significantly different measured values of cord blood acid–base parameters. In arterial cord blood, there were significant decreases of pH, standard bicarbonate (HCO_3_^–^), and base excess (BE), and significant increases of arterial carbon dioxide tension (PaCO_2_), PO_2_, and lactate from *T*_0_ to *T*_90_, with the most pronounced changes at *T*_0_–*T*_45_. Similar changes occurred in venous blood pH, HCO_3_^–^, BE, PaCO_2_, and lactate, although the changes were smaller and most pronounced at *T*_45_–*T*_90_. No significant changes were observed in venous PO_2_.

In another prospective observational study on 60 vaginally delivered healthy term newborns, Valero et al. sampled umbilical cord blood immediately after delivery or at the time when the umbilical cord pulsation spontaneously ceased. DCC was associated with a significant decrease in pH, oxygen saturation, glycemia, oxygen content, HCO_3_^–^, and BE, and an increase in lactate and PCO_2_, but without a change in PO_2_ in either artery or vein [75]. Taken together, the two studies [74, 75] observed a trend toward a respiratory and metabolic acidosis, particularly decreases in pH, BE, and HCO_3_^–^, and increases in PCO_2_ and lactate in the umbilical artery and vein, and clearly showed that the measured values of umbilical cord blood gases and lactate were sensitive to delayed sampling procedures.

However, in a study of normal vaginally delivered neonates from healthy full-term mothers randomly assigned to ECC (< 10 s) or DCC (> 2 min) group, De Paco et al. [76] reported a delay of 2 min before umbilical cord clamping did not significantly change the acid–base and gas analysis results in the umbilical vein or artery, with the exception of a higher mean umbilical artery PO_2_ value in the delayed clamping group. No significant differences in umbilical vein or artery PCO_2_ or HCO_3_^–^ values were observed between ECC group and DCC group. Moreover, they obtained the same results from the study in 2016 [70].

In consistent with these findings, Andersson et al. [71] observed that the proportion of valid blood gas samples was similar between the DCC and ECC groups, and there was no difference in umbilical artery pH or pCO_2_ between groups with the exception of a higher umbilical artery PO_2_ value in the DCC group. This finding of a higher umbilical artery PO_2_ value may be explained by the fact that the neonates started breathing while the umbilical cord was unclamped. Therefore, they [70, 71, 76] concluded that DCC did not influence umbilical blood gas findings and allowed DCC to be practiced without compromising the important data yielded by these analyses.

For ethical reasons, these studies [70, 71, 74–76] involved term newborns with a normal vaginal delivery who did not require resuscitation at birth and reported discrepant results. Nevertheless acid–base status and cord blood gas analyses were recommended in all high-risk deliveries. Evidence of normal acid–base status and gas analysis in umbilical vein and artery provided useful support if an intrapartum hypoxic–ischemic event was suspected. There was no clear conclusion whether DCC would lead to intrapartum hypoxic–ischemic event, and may be of no clinical relevance. Thus, further studies are needed to determine the effect of DCC on blood gas analysis in high-risk newborns.

## The effect of DCC on cord blood units

Cord blood is a widely accepted stem cell source, especially in the pediatric setting. Implications of DCC for public cord blood banking remained unclear. Ciubotariu et al. [77] observed that the impact of DCC on the public cord blood banking was substantial. The results of their study indicated that DCC of 30–60 s had a small negative impact on the collection of high-total nucleated cells (TNCs)-count Cord blood units (CBUs). However, increasing birth-to-clamping to more than 60 s decreased significantly both TNCs content and volume and reduced drastically the chances of obtaining clinically useful CBUs. Also, Allan et al. [78] found that the mean volume and the TNCs in units with no delay in clamping were significantly greater than those with all categories of delayed clamping including delayed by 20–60 s, more than 60 s, or more than 120 s.

Based on these observations [77, 78], the authors recommend that DCC with no more than 60 s [77] or clamping the cord at 30 s [78] may facilitate optimal infant health outcomes and still allow the collection of units with high TNC content, which rescued adequate volume of cord blood for collection, banking, and the use in clinical transplantation. However, Frändberg et al. [79] reported that although the volume of cord blood collected after DCC was significantly lower, the TNC content in units collected was not significantly lower compared to units collected after ECC. They believed DCC had no major effect on collection efficiency, which suggested that cord blood collection with delayed clamping routines was feasible.

Therefore, the effect of the different durations of DCC on the collection volume and the cell content require further study, and these strategies require further refinement, which eventually allows for sufficient placental transfusion to ensure the infant donor’s welfare and to collect the adequate volume of clinically useful cord blood.

## The effect of DCC on stem cells

Human umbilical cord blood contains significant amounts of stem and progenitor cells and is currently used in the treatment of several life-threatening diseases. DCC enhanced blood flow from the placenta to the neonate, which should increase stem cell supply to newborns [80]. However, a prospective randomized control single-center study [81] found that compared with ECC group (5–10 s), all peripheral hematopoietic progenitor cell (HPC) counts were lower in the DCC group (30–45 s). For unexpected results, the authors speculated that placental transfusion from DCC resulted in an increase in transfer of the stated placental factors, such as stem cell factor and stromal-derived factor-1 which facilitated the homing process of HPCs to the target organ receptors resulting in a decrease in HPCs values in the blood stream.

## Conclusions

In conclusion, DCC resulted in significant health benefits for term and preterm infants. DCC was not associated with any clinically significant difference in the risk of post-operative hemorrhage, neonatal hyperbilirubinemia or symptomatic polycythemia compared to ECC. DCC in preterm and term infants was feasible and safe, and there should be no hesitation in implementing this procedure routinely. Due to the side effects of DCC for 45 s affecting cord blood acid–base parameters and DCC for more than 60 s reducing clinically useful cord blood units (CBUs), further studies are needed to conduct to determine the optimal cord clamping time.
